# Facile Cellulase Immobilisation on Bioinspired Silica

**DOI:** 10.3390/nano12040626

**Published:** 2022-02-13

**Authors:** Vincenzo Lombardi, Matteo Trande, Michele Back, Siddharth V. Patwardhan, Alvise Benedetti

**Affiliations:** 1 Department of Molecular Sciences and Nanosystems, Ca’ Foscari University of Venice, Via Torino 155, 30172 Mestre, Italy; michele.back@unive.it; 2 Department of Biotechnology, University of Manchester, 131 Princess Street, Manchester M1 7DN, UK; matteo.trande@manchester.ac.uk; 3 Department of Chemical and Biological Engineering, The University of Sheffield, Mappin Street, Sheffield S1 3JD, UK

**Keywords:** immobilisation, bioinspired silica, cellulase

## Abstract

Cellulases are enzymes with great potential for converting biomass to biofuels for sustainable energy. However, their commercial use is limited by their costs and low reusability. Therefore, the scientific and industrial sectors are focusing on finding better strategies to reuse enzymes and improve their performance. In this work, cellulase from *Aspergillus niger* was immobilised through in situ entrapment and adsorption on bio-inspired silica (BIS) supports. To the best of our knowledge, this green effect strategy has never been applied for cellulase into BIS. In situ entrapment was performed during support synthesis, applying a one-pot approach at mild conditions (room temperature, pH 7, and water solvent), while adsorption was performed after support formation. The loading efficiency was investigated on different immobilisation systems by Bradford assay and FTIR. Bovine serum albumin (BSA) was chosen as a control to optimize cellulase loading. The residual activity of cellulase was analysed by the dinitro salicylic acid (DNS) method. Activity of 90% was observed for the entrapped enzyme, while activity of ~55% was observed for the adsorbed enzyme. Moreover, the supported enzyme systems were recycled five times to evaluate their reuse potential. The thermal and pH stability tests suggested that both entrapment and adsorption strategies can increase enzyme activity. The results highlight that the entrapment in BIS is a potentially useful strategy to easily immobilise enzymes, while preserving their stability and recycle potential.

## 1. Introduction

In recent years, biomass has become a sustainable source for chemical industries; biomass waste has reached about 140 gigatons per year [1,2,3]. Particularly, biomass is widely exploited for the production of biofuels (e.g., bioethanol, biohydrogen, and biodiesel) [4], among which bioethanol is the most widespread worldwide, with 142.6 billion litres produced in 2019, corresponding to 70.5% of the total biofuels share [5,6,7].

In order to increase the efficiency of the conversion process and, thus, the yields of the biofuels produced from biomass recycling, biocatalysts, such as enzymes, are usually employed. The most used enzymes belong to the cellulases family and are further divided into three main subfamilies: exo-glucanase, endo-glucanase, and β 1,4 glucosidase. The exo-and endo-glucanases work on the insoluble and soluble forms of cellulose, respectively, to produce oligosaccharides, while the glucosidase family works on oligosaccharides to produce glucose [8,9,10]. The structure of cellulases is divided into two main parts: a carbohydrate-binding domain (CBD) and a catalytic domain (CD). The CBD facilitates cellulose hydrolysis by bringing the CD in contact with the cellulose network, while the CD is the principal site where the catalytic hydrolysis occurs [11,12]. The hydrolysis mechanism is catalysed under acidic conditions, where the catalytic reaction passes through a glycosyl-enzyme intermediate, subsequently enabling the hydrolysis of the substrate [13,14].

However, cellulase high costs (corresponding to about 40% of the total costs of the cellulosic ethanol production) and drawbacks related to its use in soluble form (such as low stability, poor reusability, and limitations when used in continuous reactors [15,16]) still hinder its widespread exploitation. In this framework, immobilisation or confinement technology seems to be a good strategy to improve the catalytic features of enzymes like cellulase, especially their re-usability in different catalytic cycles [17,18,19,20]. Immobilisation techniques exploit a covalent or non-covalent bonding such as electrostatic interactions [21,22]. As far as the covalent bonding is concerned, bifunctional coupling agents are used to tether enzymes on supports [23,24]. Concerning the non-covalent bonding, electrostatic interactions, which depend on the charge of the support and the isoelectric point of the enzyme [25,26,27,28], give rise to weak bonds between the support and the enzyme.

Several techniques have been developed for the confinement of cellulases including adsorption [29], entrapment [30], encapsulation [31], and chemical complex formation [32]. Moreover, a wide range of materials have been proposed as supports for cellulase immobilisation like porous silica [33,34,35], paramagnetic particles (Fe_2_O_3_) [36,37] or cobalt ferrite nanoparticles [38], and organic polymeric materials [39].

Silica supports are widely used due to their low cost, good stability, inertness, and easy modulation of their physicochemical properties [30,31,33,40,41]. Furthermore, Fe_3_O_4_ magnetic nanoparticles have been used to immobilise cellulase [36,42,43,44,45,46]. However, the preferred pH condition to immobilise cellulase is slightly acidic, thus dissolving Fe_3_O_4_ back to Fe^2+^ and Fe^3+^. In addition, the aggregation of Fe_3_O_4_ magnetic nanoparticles decreases the mass transfer rate in the solution [47,48].

Typically, supports are synthesized under harsh conditions such as high temperature, different acid or basic conditions and long times of reaction. For instance, it is worth noting that the synthesis of SBA-15 (a popular mesoporous silica support) is resource and energy intensive and, thus, unsustainable, uneconomical, and not scalable. As such, industrial use of mesoporous supports is very limited, although they show remarkable characteristics. Recently, much attention has been paid to bio-inspired methods that are able to produce materials under green conditions and enable in situ enzyme immobilisation [49]. One of the main advantages of bio-inspired methods is that the material properties (e.g., surface area, particle size, porosity, etc.) can be fine-tuned through the use of appropriate processing parameters, additives and silica precursors. Moreover, the preparation time is short (5–10 min) and the synthesis is fully aqueous, requiring mild conditions (pH 7 and room temperature). This method uses organic additives during the silica network formation, which can act as catalysts, aggregation promoting agents or structure-directing agents, or, more probably, a combination of these. In the literature, several studies investigated the possibility of immobilising enzymes by bio-inspired silica (BIS) [50]. For instance, Forsyth et al. [51] investigated various additives such as pentaethylenehexamine (PEHA), tetraethylenepentamine (TEPA), triethylenetetramine (TETA), and diethylenetriamine (DETA) for the entrapment of the lipase enzyme into a BIS support. The authors obtained a high immobilization efficiency close to 100% and high levels of activity and stability. Polyethyleneimine (PEI) was also successfully used for enzyme entrapment, even though poor results were obtained for lipase and carboxylesterase immobilization [52,53]. Although many works report a significant loss of activity for the confined enzyme, the PEI additive appears efficient for horseradish peroxidase immobilization [54]. In addition, additives like polyallylamine (PAH or PAA), poly(amidoamine) dendrimers (PAMAM), and poly-L-lysine (PLL) can successfully immobilize enzymes, such as D-amino acid oxidase, glucose oxidase, horseradish peroxidase, and adenosine deaminase [49]. Despite the promising performances, there are few articles about the use of BIS as support for cellulase immobilisation in the literature [55].

In this framework, this work focuses on the entrapment and adsorption of cellulase from *Aspergillus niger* using silica systems synthesized via the bio-inspired method [56,57]. Due to the mild nature of the synthesis, enzymes can be entrapped in situ into the BIS pores, thereby allowing a one-step process of confinement. Although this method has been successfully applied for various enzymes [51,58], it has never been used for cellulase. Similar to more traditional silicas, enzymes can be immobilized on BIS via adsorption by exploiting the surface functionalities. In order to assess the performance of cellulase immobilization on BIS, we compared the in situ entrapment with other post-synthetic adsorption strategies reported in the literature [27,31,33,48,59,60,61]. Moreover, to explore different porosities and surface chemistries, different BIS samples were synthesized by varying the synthesis conditions including the additives used and the pH of the post-synthesis treatment [62]. Five different additives and different final pH (7, 5, and 2) were used for the synthesis of the supports. In particular, additives containing a different number of secondary amines were chosen in order to investigate their interactions with proteins during both entrapment and adsorption confinement. Preliminary tests were performed using bovine serum albumin (BSA) in order to choose the better conditions for both entrapment and adsorption. Afterwards, cellulase was immobilized on BIS using the optimized conditions and its activity was analysed along with the effects of operational pH and the thermal stability of the systems. Finally, the catalytic activity of the samples was compared with the performances of both the enzymes confined on silica networks prepared with different methods and the ones confined on other inorganic and organic supports.

## 2. Materials and Methods

### 2.1. Materials

Sodium metasilicate pentahydrate 95% (71746), diethylenetriamine (DETA) 99% (D93856), triethylenetetramine (TETA) 97% (90460), pentaethylenehexamine (PEHA) technical grade (292753), poly(allylamine hydrochloride) (PAA) average Mw 50,000 (283223), polyethyleneimine (PEI) average Mw~800 by LS, average Mn~600 by GPC (408719), hydrochloric acid 37% (1.00317), 3,5-dinitrosalicylic acid 98% (128848), sodium hydroxide 98% (S5881), potassium sodium tartrate tetrahydrate 99% (217255), phenol ≥ 99% (W322318), sodium sulfite ≥98% (S0505), carboxymethylcellulose sodium salt low viscosity (C5678), sodium citrate dihydrate ≥99% (W302600), and citric acid monohydrate ≥99% (C1909) were obtained from Sigma Aldrich, Milan, Italy. The proteins, cellulase from *Aspergillus niger*~0.8 U/mg (22178, labelled as Cell_EG) and bovine serum albumin (BSA) heat shock fraction, pH 7, ≥98% (A9647) with Bradford reagent for 0.1–1.4 mg/mL protein (B6916), sodium phosphate monobasic ≥99% (S0751), and sodium phosphate dibasic ≥98% (04276), from Sigma Aldrich, Milan, Italy. The D-(+) glucose analytical standard ≥99.5% (G8270), trizma base 99.9% (T1503), dodecyl sulfate sodium salt ≥99% (436143), β-mercaptoethanol ≥99% (M3148), and bromophenol blue indicator (1.08122) from Sigma Aldrich, Milan, Italy. The broad range standards marker unstained SDS-PAGE standards, broad range, 200 µL (1610317) from Bio-Rad, Milan, Italy. All reagents were used without further purification. Except for cellulase protein, which was purified with SEC analysis as described in Section S2.4.

### 2.2. Synthesis Bio-Inspired Silica Supports

The BIS synthesis was performed in a 100 mL beaker glass by first preparing a 40 mL solution of sodium metasilicate pentahydrate (0.64 g) in deionized water. In a test tube, desired amount of the selected additive (DETA, TETA, PEHA, PEI, and PAA) was weighed and was dissolved in 20 mL deionized water. The two solutions were mixed to reach a final [Si] of 30 mM. For DETA, TETA, and PEHA, the final [Si]: [N] was 1, while PEI and PAA were used at a final concentration of 1 mg/mL. The reaction mixture was neutralized by adding a pre-determined amount of 1 M HCl under stirring at room temperature to reach the final pH of 7 ± 0.05 after 5 min. The as-synthesised samples were termed “pH7” materials. We have recently shown that post-synthetic acidification of the precipitated BIS leads to partial removal of the additives, leading to increased porosity and surface chemistry changes. As such, “pH5” and “pH2” samples were prepared by a post-synthetic treatment in a second step by further addition of a pre-determined amount of 1 M HCl to reach the desired pH value (pH5 or pH2) after other 5 min. After the reaction (either one-step to get pH7 samples with a total reaction time of about 5 min or two-step to get pH5 and pH2 samples with 10 min of total time reaction), the suspensions were centrifuged at 3000× *g* for 15 min and washed three times with deionized water. After washing, the pellet was dried at room temperature.

### 2.3. Materials Characterization

BIS characterization was performed before and after both entrapment and adsorption of the proteins. The particle size and morphology were evaluated with a Zeiss Sigma VP FEG-SEM, Milan, Italy. The surface area and the porosity were analysed by N_2_ physisorption analysis using Micromeritics ASAP 2010, Milan, Italy. The protein quantification and the catalytic activity were determined by UV-visible analysis with Agilent 8453 UV-visible spectrophotometer, Milan, Italy by measuring the absorbance at 595 nm (for protein quantification) and 500 nm (for cellulase assay). The qualitative analysis of entrapment and adsorbed systems were recorded by Diffuse Reflectance Fourier Transform Infrared Spectroscopy (DRIFT-IR), Milan, Italy with a NEXUS-FT-IR instrument implementing a Nicolet AVATAR Diffuse Reflectance accessory, Milan, Italy.

### 2.4. Protein Adsorption Method

In a first tube, a protein stock solution was prepared at 10 mg/mL in distilled water. In a second tube, 10 mg silica samples were suspended in 1 mL of distilled water with pH~7. These two solutions were mixed to a final volume of 2 mL and the final concentration of the support was 5 mg/mL and the protein concentration was 1 mg/mL. The samples were incubated for 2 h under stirring in a water bath at room temperature. The samples were then centrifuged at 3000× *g* for 15 min and washed three times with distilled water; each supernatant was collected in order to quantify free protein remaining.

### 2.5. Protein Entrapment Method

For the entrapment of proteins, during BIS synthesis described in Section 2.2, protein solution was added immediately during the neutralization with 1 M HCl and the resulting solution was gently mixed. The final protein concentration was 1 mg/mL. As described in Section 2.2, the reaction mixture was left unstirred for 5 min and centrifuged, washed, and dried. The supernatants were stored after each round of washing in order to quantify free protein remaining.

### 2.6. Protein Quantification

The quantification of protein loading was carried out by analysing the supernatants recovered after the reaction and were analysed by the Bradford assay [63]. The assay was performed by adding a predetermined amount of Bradford reagent to either the supernatant samples or calibration samples in cuvettes. The calibration curve (see Figure S6) was prepared using the BSA in a range of 0.1–1.2 mg/mL. The final solution was mixed three times and the colour was left to develop for 15 min. The sample absorbance was recorded at 595 nm using distilled water as blank. As the protein–dye complex is stable up to 60 min, the absorbance was recorded before 60 min and after 15 min of the Bradford reagent addition. For each calibration, the averages of the three different measurements were reported. The amount of the protein loaded during both entrapment and adsorption was calculated using the calibration curve obtained. The enzyme loading efficiency (%) was determined as follows (Equation (1)):

(1) $$Loading\;efficiency\;\left(\%\right)=\frac{E_{tot}-\left(E_{recovery}+W1+W2+W3\right)}{E_{tot}}\times 100\;$$

where *E_tot_* is the initial enzyme quantity present in the mixture (during entrapment or adsorption); *E_recovery_* is the amount of enzyme in the supernatant immediately after entrapment or adsorption; and *W*_1_, *W*_2_, and *W*_3_ are the amounts of enzymes present in the supernatant after each washing solution. For each sample, the measurement of the loading efficiency was performed three times.

### 2.7. Cellulase Activity Assay

The enzyme activity was evaluated through the analysis of the reducing sugar formed in the solution after the catalytic action of the enzyme using carboxymethylcellulose sodium salt (CMC) as substrate. The concentrations of the reducing sugar were analysed by the reaction with 3,5-dinitrosalicylic acid (DNS) reagent [64,65]. The method following the work of T. K. Ghose et al. [64]. In a tube, 0.05 mL of protein solution (0.2 mg/mL of the cellulase in 0.05 M citrate buffer at pH 4.8) and 0.05 mL of CMC solution (2% *w/v* CMC in 0.05 M citrate buffer) were mixed at 50 °C for 30 min. Then DNS reagent (0.3 mL) was added. The final solution was boiled for 10 min and then immediately transferred to a cold-water bath and 1.6 mL of distilled water was added. The colour formed was monitored at a wavelength of 500 nm. A calibration curve with glucose standard in a range of 0.25–1 mg/mL was used to evaluate the amount of the reducing sugar produced in the solution. The relative activity (RA%) of the different confinement systems and their catalytic cycles (RACC%-Relative activity of the catalytic cycles) were calculated following Equations (2) and (3):

(2) $$RA\;\left(\%\right)=\frac{Rg_{ce}}{Rg_{fe}}\;\times \;100\;$$

(3) $$RACC\;\left(\%\right)=\frac{Cg_{ce}}{Rg_{ce}}\;\times \;100\;$$

where *Rg_ce_* is the amount of reducing glucose from enzyme confined (mg/mL), *Rg_fe_* is the amount of reducing glucose from free enzyme (mg/mL), and *Cg_ce_* is the amount of glucose from system reused (mg/mL). In each recycling step, the samples were washed three times with 1 mL of distilled water and centrifuged at 3000× *g* for 15 min, in order to remove the hydrolysed product after each catalytic cycle. For each sample, the measurement of the enzyme activity was performed three times.

## 3. Results and Discussion

### 3.1. Analysis of the Synthesized BIS Supports

The bio-inspired method was used as an alternative green process to produce silica supports in a water environment without organic solvents. BIS was carried out in water solution using a sodium silica precursor (thus, avoiding alkoxysilanes), in the presence of an additive (usually an amine or polyamine) at room temperature. Different BIS were prepared by changing the additive (DETA, TETA, PEHA, PEI, and PAA) and the post-synthesis pH as reported in Table 1. 

**Figure 1:**
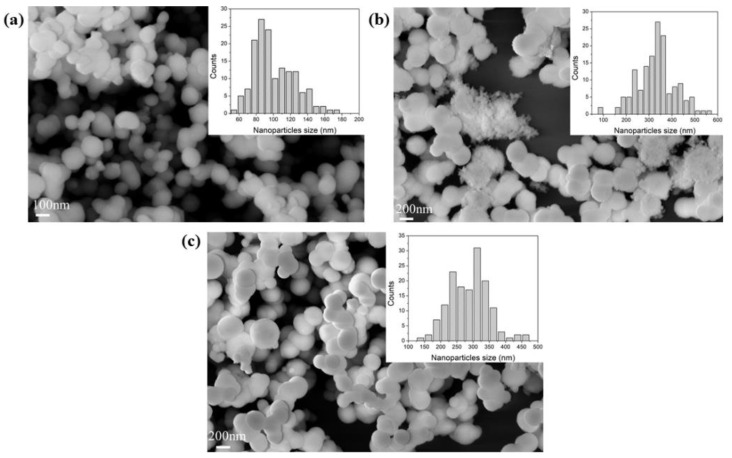
FE-SEM images of sample (**a**) BIS-TETA _7, (**b**) BIS-TETA _5, and (**c**) BIS-TETA _2. Insets show particle size distributions.

The porous structure and morphology of the samples were analysed by nitrogen physisorption and SEM analyses; meanwhile, the chemical composition was probed using FTIR-DRIFT. For each sample, the measurements were performed three times. The results can provide useful information on partial or full removal of the additive from the supports. All the samples, synthesized with different additives, showed a certain degree of aggregation of the particles, as shown in Figure 1 and Figure S1. The particle sizes were typically between 100 and 400 nm, all consistent with the literature [51,58].

**Figure 2:**
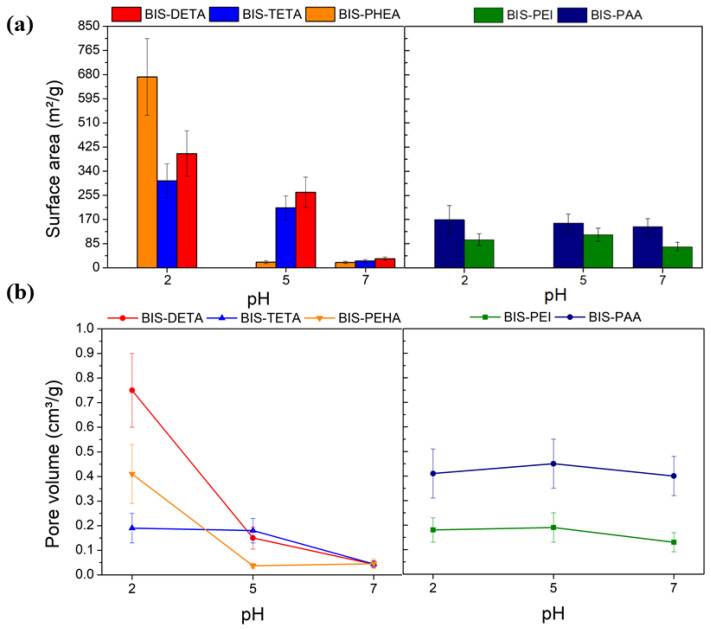
N_2_ physisorption analysis results: (**a**) Surface area estimated by BET analysis and (**b**) pore volume as a function of the pH.

According to the IUPAC classification [66], the nitrogen adsorption isotherms obtained were of type II and III, typical of macroporous materials (see Figure S2). Figure 2 and Table S1 show the values of the specific surface area and pore volume of the samples. At pH 2, the specific surface area of samples synthesized by DETA, TETA, and PEHA are higher than the values obtained at pH 7 and 5 (Figure 2a). These differences are related to the removal of the additives from the support after the treatment at pH 2, consistent with previous reports [62]. This difference is also supported by the increased pore volume of supports treated at pH 2, as indicated in Figure 2b. Additional evidence of the removal of the additive from the supports synthesized at pH 2 is provided by the FTIR analysis as shown in Figure S3. The supports synthesized with PAA and PEI at pH 2, 5, and 7 do not show evident differences from both gas adsorption and FTIR analysis, potentially due to their entanglement and the consequent resistance to removal.

### 3.2. Confinement of the Proteins

The protein immobilisation was carried out by both adsorption and entrapment method. First, cellulase was purified by size exclusion chromatography to remove protein aggregates and impurities (the methods and analysis of the cellulase purified are reported in Section S2.1 and Figure S5 of the Supplementary Information). The adsorption method on different supports was performed at pH 7 in water at room temperature. The protein entrapment was carried out in situ with a one-pot step reaction at pH 7 (obtaining the protein caged into the silica network). During the entrapment process, proteins were not exposed to pHs different to 7 to avoid protein instability.

At first, the adsorption and the entrapment methods were carried out by employing BSA (Figure S4a,b) to select the best support with maximum protein loading before cellulase enzyme immobilization (Figure S4c,d).

#### 3.2.1. Protein Adsorption

Adsorption was performed for 2 h, at room temperature, pH 7, using water as solvent. As supports were used BIS prepared at pH 2, 5, and 7. For BIS prepared at pH 7, the additives are completely entrapped in the silica network, resulting in a functionalized surface of the support. Instead, for the BIS treated at pH 2, the amines are soluble, and they can be eliminated from the silica network by washing, while the polyamines, due to their size and their closer interaction with the silica network, are not completely eliminated by washing. For the BIS prepared at pH 5, an intermediated behaviour is expected.

Tables S2 and S3 show the quantities of the proteins loaded and the samples used during the tests.

**Figure 3:**
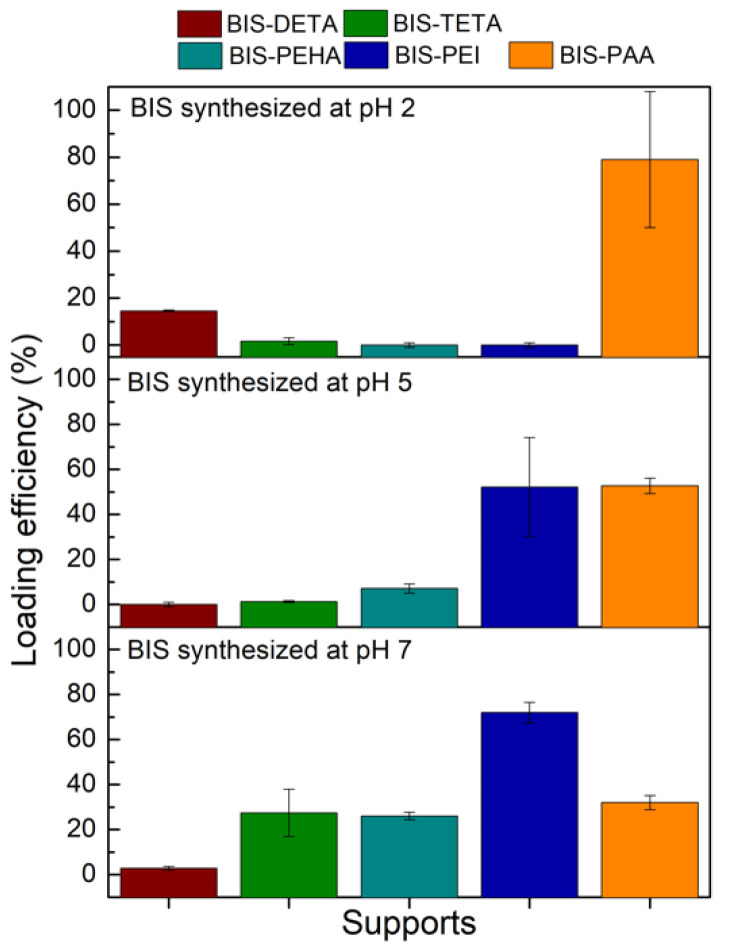
BSA loading efficiency (%) for the different BIS supports.

In order to select the best pH for each additive (DETA, TETA, PEHA, PEI, and PAA) for further cellulase adsorption, the loading efficiency achieved with BSA protein was firstly considered (Figure 3). BIS-DETA and BIS-PAA synthesized at pH 2 and BIS-TETA, BIS-PEHA, and BIS-PEI synthesized at pH 7 show the highest loading efficiencies. The additives (ammine and polyamine) are still present in the samples prepared at pH 7, revealing a certain affinity related to the interaction between the negative charges of the protein and the positive charges of the additives.

For BIS-DETA and BIS-PAA synthesized at pH 2, we can consider that: (a) the absence of the additives increases the development of the porosity (pore volume and surface area) and the surface chemistry of the support influences the adsorption amount of the protein (sample BIS-DETA_2); (b) when the additives are not completely removed, (lower value of pore volume and surface area) a positive charged surface can be present (sample BIS-PAA_2). The latter consideration applies also to the three samples prepared at pH 7, where the additives are still present in the pore structures. Irrespective of the loading strategy employed, the BIS-PAA sample ensures the best loading efficiency.

**Figure 4:**
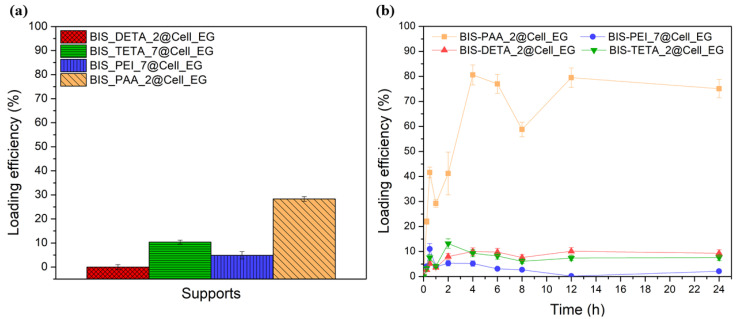
(**a**) Loading efficiency % after 2 h of Cell_EG adsorption and (**b**) adsorption kinetics of the Cell_EG.

The supports with the best BSA adsorption performance, excluding BIS-PEHA with a performance very close to BIS-TETA and for this reason not considered, were chosen also for the cellulase enzyme loading analyses. Keeping the same adsorption conditions of BSA, the cellulase loading is lower than BSA (see Figure 4a). BIS-PAA_2@Cell_EG shows the best loading efficiency (28 ± 1%). The adsorption kinetics (see Figure 4b) describe different equilibrium states of the protein with the supports.

At the initial adsorption stages, the amount of cellulase is relatively low for all the supports, but after 1 h the adsorbed amount begins to increase. The increase is much larger for the BIS-PAA_2 support. The cellulase continues to be absorbed as long as the surface is completely filled (see the yellow line with square in the Figure 4b). After 24 h, the adsorption curve of the protein shows an asymptotic behaviour.

#### 3.2.2. Protein Entrapment

**Figure 5:**
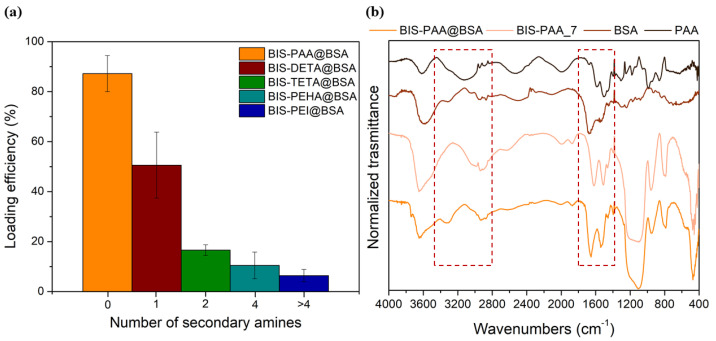
(**a**) Loading efficiency for BSA with different additives. (**b**) FTIR spectra of BIS-PAA@BSA, BIS-PAA_7 (system without BSA), protein powder (BSA), and polyamine powder (PAA).

During the reaction, the protein is likely to interact with the additive by charge attractions due to the different isoelectric point of the proteins (Ip _(BSA)_ = 4.9 and Ip _(cellulase)_ = 4.8) with respect to the additives (9 < Ip < 12). Then, proteins and additives interact with SiO^−^ moieties on the surface of the reacting silicate species [67]. These interactions lead to the entrapment of both the protein and the additive in BIS. The best loading efficiency of about 87 ± 7% for BSA is obtained with PAA additive, as shown in Figure 5a (see also Table S4 of the Supplementary Information for the errors). This result is likely due to the strong interaction between the polyelectrolyte (PAA) and BSA protein forming a BSA–PAA complex during entrapment [68]. The binding between BSA and PAA is not only associated to Coulombic interactions, but it is also due to thermodynamic effects (loss of chain conformational entropy due to interaction and release of the counterions, Na^+^ or Cl^−^ in this case, during the complex formation of the protein/polyelectrolytes) [69,70]. Indeed, as the number of secondary amines increases (PEI > PHEA > TETA > DETA), the protein loading decreases confirming the importance of the additive-protein interactions during entrapment. Figure 5b shows the FTIR-DRIFT spectra of BIS-PAA@BSA, BIS-PAA_7 (support without BSA), BSA protein powder and polyamine powder. The signals at 3280, 1650–1550, and 1400–1200, cm^−1^ are attributed to entanglement between protein and additives used during the entrapment. Peaks at 1400–1200 cm^−1^ are due to the combination of the NH bending with the C–N stretching vibration with small contributions from the C–C stretching vibration. The 1650–1550 cm^−1^ peaks are mainly due to the stretching of the double bond C=O and C–N bond, whereas the 3280 cm^−1^ band represents the NH stretching vibration. However, the intensity of the signals (in particular, at 1400–1200 and 3280 cm^−1^) can be affected by the presence of polyamine into the support. The other peaks at 3600 cm^−1^ represent the Si-OH and water associated with silica, while the peaks between 1100 and 800 cm^−1^ are from stretching and bending of the Si–O–Si.

The entrapment systems with the best BSA performance (BIS-DETA@BSA, BIS-TETA@BSA, BIS-PAA@BSA) were used also for the entrapment of cellulase. A maximum loading of ~30% was obtained with the BIS-PAA support. Overall, a lower loading of cellulase was observed compared with BSA loading (see Table S4 of the Supplementary Information).

### 3.3. Catalytic Activity of Immobilised Cellulase

**Figure 6:**
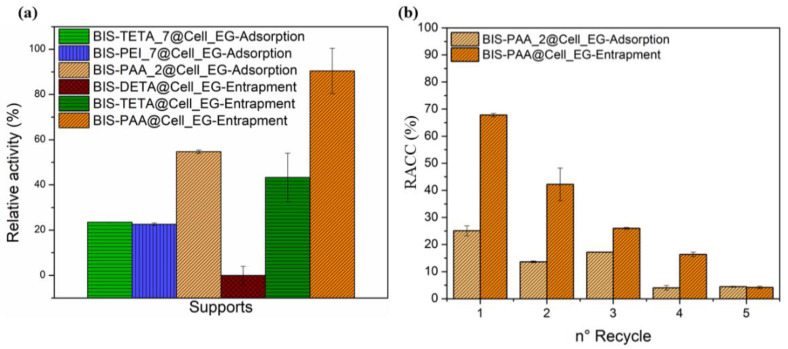
(**a**) Relative activity (%) of the different systems and the (**b**) residual activity after the five cycles.

The cellulase activity was investigated by the Endoglucanase assay that produces reducing sugars by sodium carboxymethyl cellulose (CMC) hydrolysis [65,71]. The reducing sugars was evaluated by the DNS method consisting of a redox reaction between the 3,5-dinitro salicylic acid (DNS) and the reducing sugars (see Figures S7 and S8). The reducing power of these sugars derives from their carbonyl group, which can be oxidized to carboxyl group by mild oxidizing agents, whereas the DNS (yellow colour) is reduced to 3-amino-5-nitrosalicylic acid (red/brown colour). The intensity of the colour is proportional to the concentration of the sugars in the solution [65]. BIS-PAA@Cell_EG (entrapment) and BIS-PAA_2@Cell_EG (adsorption) show the best performance (see Figure 6a and Table S5), but the entrapment method displayed higher efficiency maintaining more residual catalytic activity after the immobilisation process. One of the advantages of the immobilised systems is the possibility of recycling the enzyme after the catalytic process. In order to verify this possibility, the immobilised enzyme systems were recycled for a maximum of five times for the two best performing systems (Figure 6b). Cellulase immobilised via adsorption shows nearly 50% reduction in residual activity after two cycles, which further reduced to only 5% after five cycles. The entrapped enzyme also showed a reduction in activity (from nearly 70% to 40–45%) after two cycles, with further reduction to 5% after five cycles.

**Figure 7:**
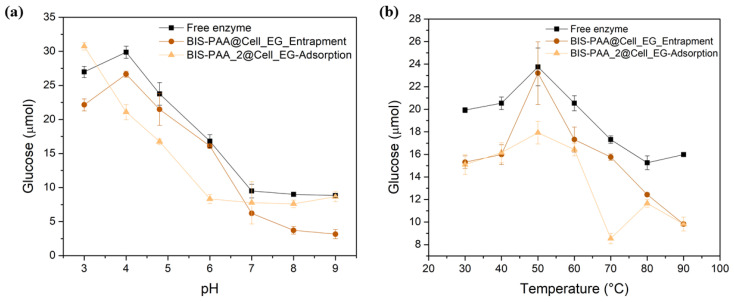
Analysis of the stability at different values of (**a**) pH and (**b**) temperature of the cellulase entrapped and adsorbed.

While the reduction in activity for adsorbed enzymes is generally attributed to leaching/loss of enzyme, the reduction in activity of the entrapped enzyme should not be related to this effect as reported in literature [50,51]. It is, thus, likely that the loss of activity after reuse is due to the unfolding or inactivation of the enzymes. The effect of different pH and temperature on enzyme stability and hence their activity is shown in Figure 7. By varying pH, entrapped enzymes show a slightly lower activity than the free enzyme, even though the trend is similar. The adsorbed system shows a different trend, but mostly has a lower activity than the free enzyme. Similarly, the thermal stability of the immobilised systems is always lower than the one of the free enzyme.

Based on the results, the entrapment method using silica bioinspired can be a fast and green synthetic procedure to confine cellulase, maintaining its catalytic activity comparable to free enzymes. Table 2 compares different silica systems used to immobilize different cellulase enzymes with our results confirming the potential of the procedure here proposed. We can also conclude that our entrapment system shows performance comparable to other materials already studied such as magnetic nanoparticles (residual activity after confinement between 90 and 95%) [36,43,45], titania (residual activity of about 75%) [72], and organic polymers (with a residual activity >90%) [39].

## 4. Conclusions

In summary, this work investigated the potential of the adsorption and entrapment methods to confine bovine serum albumin and cellulase by means of bioinspired silica. Bioinspired silica was prepared in an aqueous solution, at room temperature, using sodium metasilicate allowing the incorporation of additives (such as amines or polyamines) into the silica network. The best loading efficiency was obtained with polyamine PAA for both BSA and Cell_EG, suggesting a better affinity between PAA and the proteins. Similarly, for cellulase immobilised via adsorption, BIS-PAA_7 evidenced a good loading efficiency. Cellulase maintained about 90% of the initial activity using the entrapment method (BIS-PAA@Cell_EG), while for the adsorption (BIS-PAA_2@Cell_EG), the residual activity was 54%. The catalytic activities of the immobilised cellulase systems were tested at different pH values, temperature, and different cycles. For pH stability, the entrapment has similar activity to free enzyme, even if slightly lower. For the adsorbed systems, the activity is higher or comparable to the free enzyme at pH = 3 and pH = 9. The confined systems can be reused for five cycles: the entrapment method maintains the activity of about 40–45% after two cycles, but the residual activity after the last cycle is lower than 5%. In conclusion, we demonstrated that the entrapment via bio-inspired method, once optimised, could be a very promising technique for future developments combining an easy preparation and a green method with the preservation of the catalytic activity and stability of the cellulase enzyme.

## Figures and Tables

**Table 1:** Lists the support samples synthesized by means of the bioinspired method with different additives and at different final pH (pH 2 and 5 refer to post-synthesis treatments).

Additives	Final pH		
	7	5	2
DETA	BIS-DETA_7	BIS-DETA_5	BIS-DETA_2
TETA	BIS-TETA_7	BIS-TETA_5	BIS-TETA_2
PEHA	BIS-PEHA_7	BIS-PEHA_5	BIS-PEHA_2
PEI	BIS-PEI_7	BIS-PEI_5	BIS-PEI_2
PAA	BIS-PAA_7	BIS-PAA_5	BIS-PAA_2

**Table 2:** Comparison of different silica systems used to immobilize cellulases.

Enzyme	Support Material	Reaction Condition	Immobilisation Techniques	Activity Enzyme after Immobilisation (%)	Reference
Cellulase from *Robillarda* sp. and Cellulase from *Trichoderma reesei*	Silica fumed (commercial support)	n.a	Adsorption	42–48	[59]
			Covalent bond (R-NH_2_+Glu)	24	
			Covalent bond (R-NH_2_+Carbodiimide)	18.8	
Cellulase from *Trichoderma reesei*	Silica particles (14 nm mean size)	n.a	Adsorption	35	[33]
			Covalent bond (R-NH_2_+Glu)	25	
Cellulase	SBA-15 (Particle size ~200–250 nm, pore size = 8.9 nm, >700 m^2^g)	Acid condition 35–60–80 °C for 20 h + calcination	Encapsulation	65	[31]
Endoglucanase	FDU-12	Acid condition 160 °C for 72 h for hydrothermal treatment + acid purification	Adsorption	75.3	[35]
	FDU-12@APTES			15.6	
	FDU-12@VTMS (three-dimensional mesoporous material with pore size ~10 nm)			80.3	
Cellulase 1 from *Trichoderma reesei* Cellulases 2 which originated from *Aspergillus niger*	SiO_2_ non-porous (Fumed silica)- S1	n.a	Adsorption	>90	[60]
	SiO_2_-porous (Davisil chromatographic silica 633N)-S2			~60	
Cellulase from *Aspergillus niger*	MSN-3.8 nm	80–90 °C for 48 h in water solution + calcination	Adsorption	63.3	[61]
	MSN-17.6 nm			26.6	
	MSN-25 nm			35.8	
	MSN-200 nm			13.5	
Cellulase from *Aspergillus niger*	Bio-inspired silica (BIS) Particle size~150 nm	Neutral pH, room temperature for 5 min of the reaction	Entrapment	90	This work

## Data Availability

The data presented in this study are available on request from the corresponding author.

## Supplementary Materials

The following supporting information can be downloaded at: https://www.mdpi.com/article/10.3390/nano12040626/s1. The Supporting Information is available free of charge. Figure S1. SEM images and dimensional analysis of the different supports (a) BIS_DETA pH 7, (b) BIS_DETA pH 5, (c) BIS_DETA pH 2, (d) BIS_PEHA pH 7, (e) BIS_PEHA pH 5, (f) BIS_PEHA pH 2, (g) BIS_PEI pH 7, (h) BIS_PEI pH 5, (i) BIS_PEI pH 2, (j) BIS_PAA pH 7, (k) BIS_PAA pH 5, (l) BIS_PAA pH 2; Figure S2. Different BIS isotherms (a) BIS_DETA samples, (b) BIS_TETA samples, (c) BIS_PEHA samples, (d) BIS_PEI samples, (e) BIS_PAA samples; Figure S3. FTIR-DRIFT analysis of the (a) BIS-DETA, (b) BIS-PAA samples; Figure S4. BSA and Cell_EG surface charge distribution. Molecular surface charge distribution of BSA, PDB id: 4F5S, (a,b) and cellulase, PDB id: 1KS5, (c,d). The potential distribution was generated and visualized with UCSF Chimera; Figure S5. (a) Chromatogram of the Cell_EG after second time purification, (b) SDS-PAGE gel of the Cell_EG after first and second time purification; Figure S6. Calibration curves for the Brandford assay; Figure S7. Scheme of the reducing sugar process by DNS method; Figure S8. Calibration curve (a) Spectrum after DNS reaction with glucose standard (b) Construction of the calibration curve; Tables: Table S1. N_2_ physisorption analysis of the different support synthesized; Table S2. Morphological features and BSA loading for different BIS systems Table S3. Morphological features and cellulase loading for different BIS systems. Table S4. Different additives used and protein loading for entrapment method Table S5. Loading efficiency (%) and relative activity (%) for both cellulose adsorbed and entrapped. Reference [64,73,74] are cited in Supplementary Materials.

## Abbreviations

BIS: bioinspired silica; DETA, diethylenetriamine; TETA triethylenetetramine; PEHA, pentaethylenehexamine; PAA, poly (allylamine hydrochloride); PEI, polyethyleneimine; Cell_EG, cellulase(endoglucanase); BSA, Bovine serum albumin; CMC, carboxymethyl cellulose; DNS, 3,5- dinitro salicylic acid; FTIR, Fourier transform infrared; pI, isoelectric point; SEM scanning electron microscope; SDS–PAGE, sodium dodecyl sulphate polyacrylamide gel electrophoresis analysis; SEC, size exclusion chromatography.
