# Supplementary material for: Facile Cellulase Immobilisation on Bioinspired Silica
Source: Nanomaterials (Basel). 2022 Feb 13;12(4):626. doi: 10.3390/nano12040626 (PMC8880491; doi:10.3390/nano12040626)
Supplement: Supplementary file 1 [file nanomaterials-12-00626-s001.zip › nanomaterials-1555074-supplementary.pdf]

nanomaterials
MDPI

Supplementary Materials

Facile Cellulase Immobilisation on Bioinspired Silica

Vincenzo Lombardi [1,*], Matteo Trande [2], Michele Back [1], Siddharth V. Patwardhan [3,*] and Alvise Benedetti [1,*]

[1] Department of Molecular Sciences and Nanosystems, Ca' Foscari University of Venice, Via Torino 155, 30172 Mestre, Italy; michele.back@unive.it
[2] Department of Biotechnology, University of Manchester, 131 Princess Street, Manchester M1 7DN, UK; matteo.trande@manchester.ac.uk
[3] Department of Chemical and Biological Engineering, The University of Sheffield, Mappin Street, Sheffield S1 3JD, UK
* Correspondence: vincenzo.lombardi@unive.it (V.L.); s.patwardhan@sheffield.ac.uk (S.V.P.); benedett@unive.it (A.B.); Tel.: +44-114-222-7593 (S.V.P.); +39-041-234-6744 (A.B.)

1. Analysis of the bis supports

In Table S1 is reported the N_2 physisorption analysis of the different support synthesized.

Table S1. N_2 physisorption analysis of the different support synthesized.

Sample*	Surface area (m²/g)	Pore volume (cm³/g)
BIS_DETA_7@BSA	32 ± 4.80	0.043 ± 0.01
BIS_DETA_5@BSA	266 ± 40	0.15 ± 0.02
BIS_DETA_2@BSA	402 ± 60.34	0.75 ± 0.10
BIS_TETA_7@BSA	24 ± 3.63	0.044 ± 0.01
BIS_TETA_5@BSA	211 ± 31.72	0.18 ± 0.02
BIS_TETA_2@BSA	306 ± 46.00	0.19 ± 0.03
BIS_PEHA_7@BSA	19 ± 2.80	0.044 ± 0.01
BIS_PEHA_5@BSA	20 ± 3.00	0.18 ± 0.03
BIS_PEHA_2@BSA	672 ± 100.85	0.19 ± 0.03
BIS_PEI_7@BSA	75 ± 11.30	0.13 ± 0.02
BIS_PEI_5@BSA	117 ± 17.65	0.19 ± 0.03
BIS_PEI_2@BSA	100 ± 15.00	0.18 ± 0.02
BIS_PAA_7@BSA	145 ± 21.75	0.4 ± 0.06
BIS_PAA_5@BSA	159 ± 23.85	0.45 ± 0.07
BIS_PAA_2@BSA	170 ± 25.50	0.41 ± 0.06

* For each sample, the phisisorption measurement was performed three times.

In Figure S1 are shown the SEM images of the morphologies and dimensional analysis of the different supports synthetised.

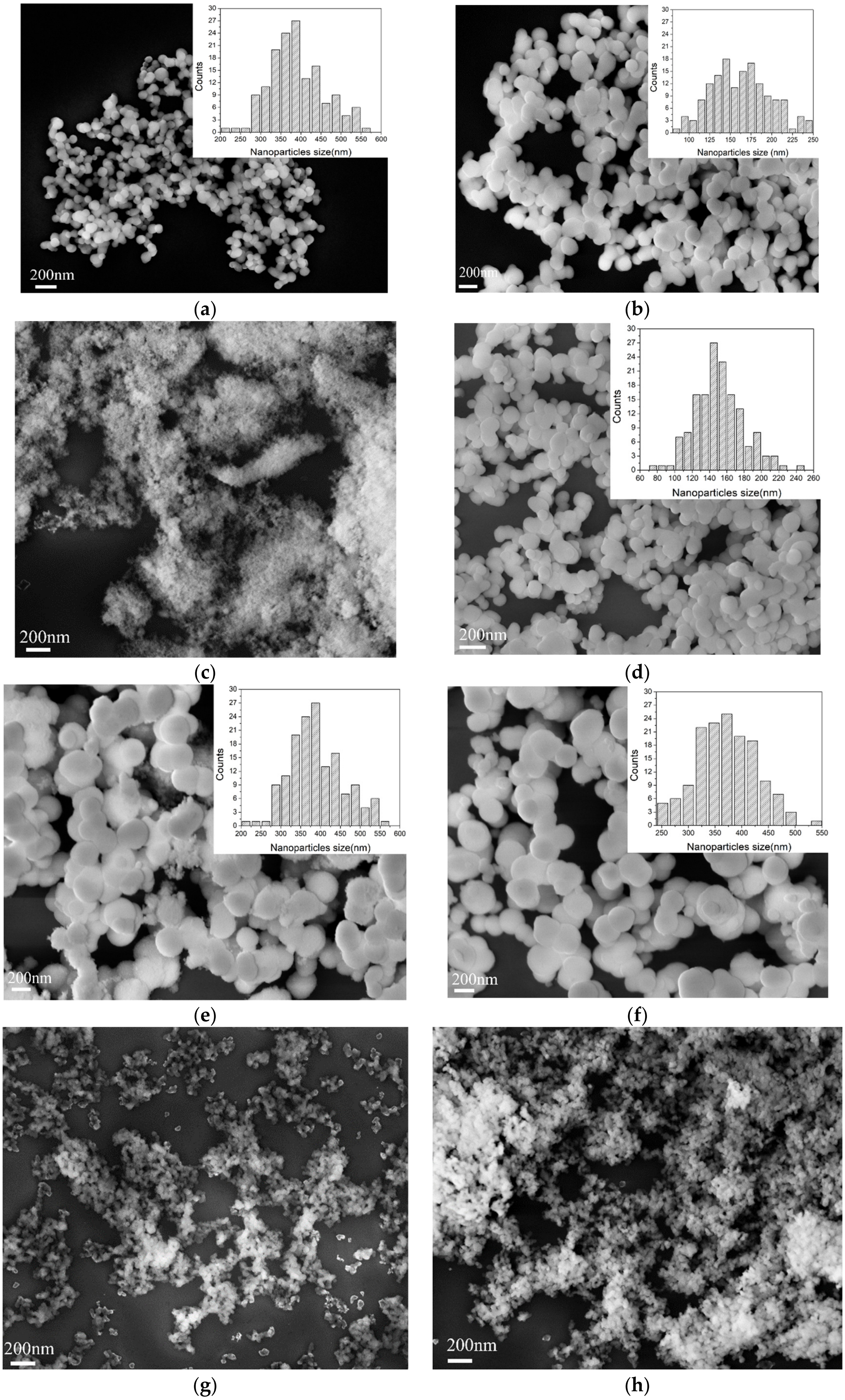
200nm
Counts
Nanoparticles size(nm)
Nanoparticles size (nm)
(a)
(b)
(c)
(d)
(e)
(f)
(g)
(h)

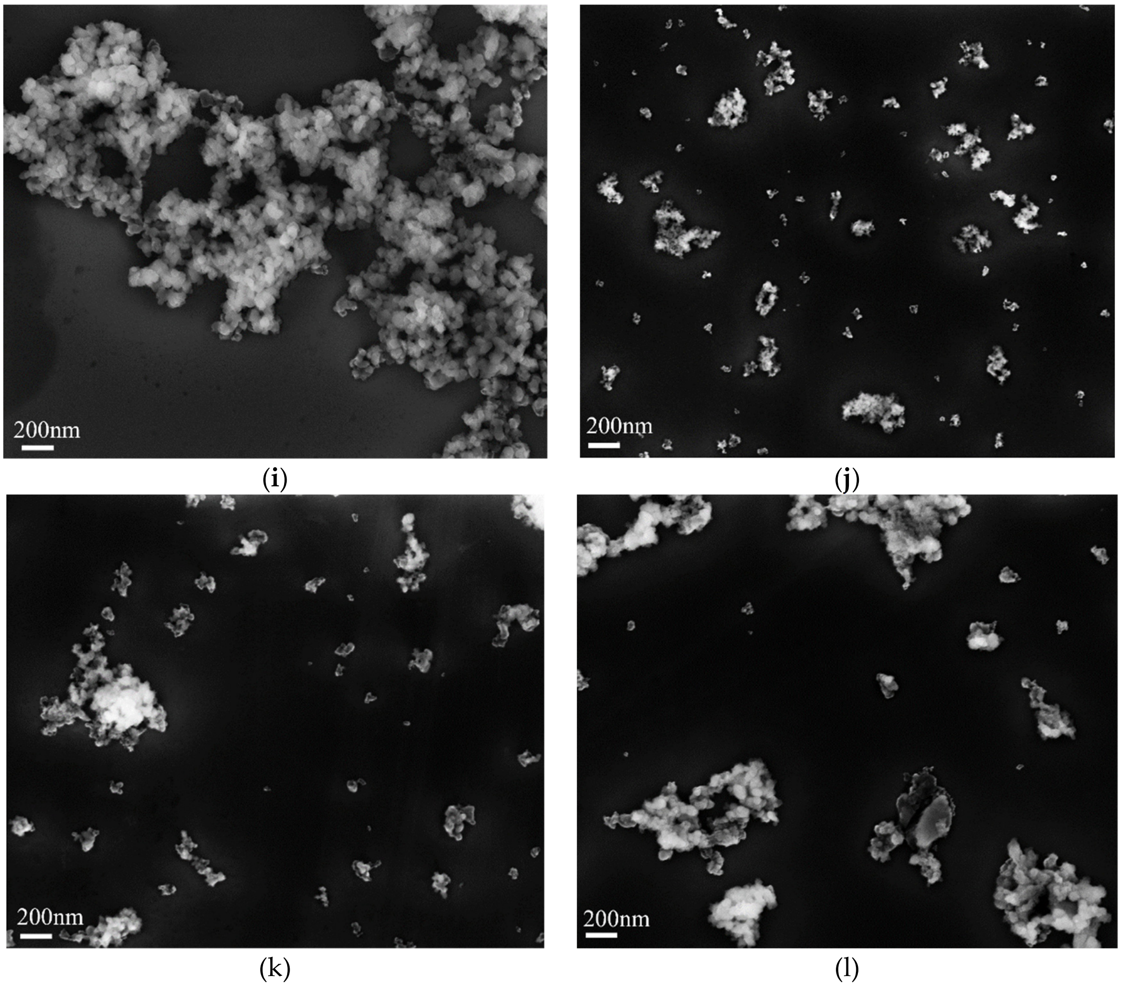

Figure S1. SEM images and dimensional analysis of the different supports (**a**) BIS_DETA pH 7, (**b**) BIS_DETA pH 5, (**c**) BIS_DETA pH 2, (**d**) BIS_PEHA pH 7, (**e**) BIS_PEHA pH 5, (**f**) BIS_PEHA pH 2, (**g**) BIS_PEI pH 7, (**h**) BIS_PEI pH 5, (**i**) BIS_PEI pH 2, (**j**) BIS_PAA pH 7, (**k**) BIS_PAA pH 5, (**l**) BIS_PAA pH 2.

In Figure S2 are reported the isotherms of all supports synthesized.

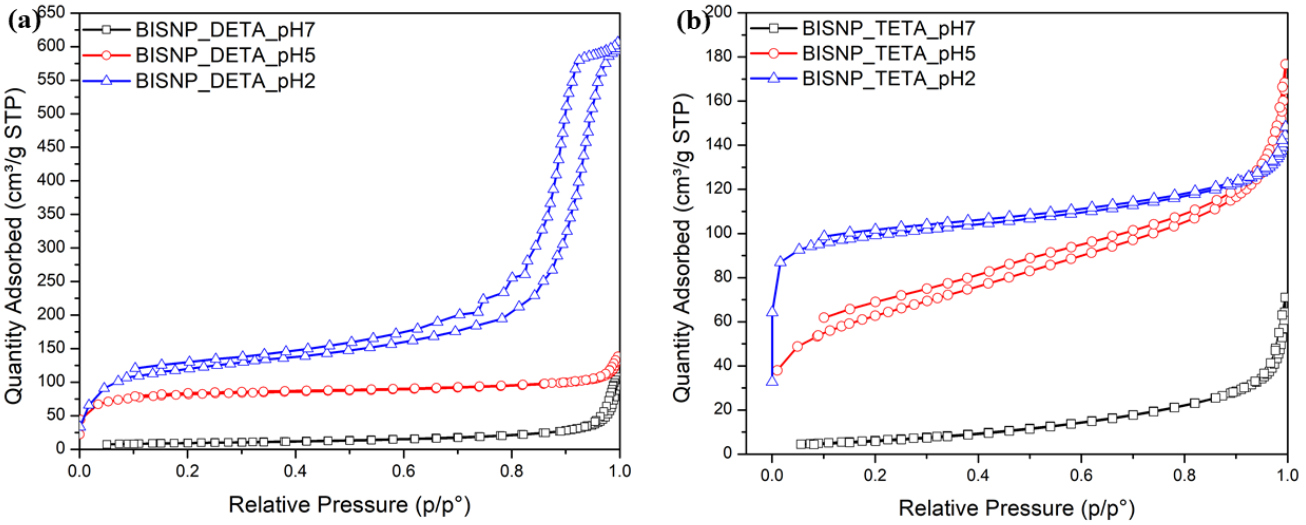

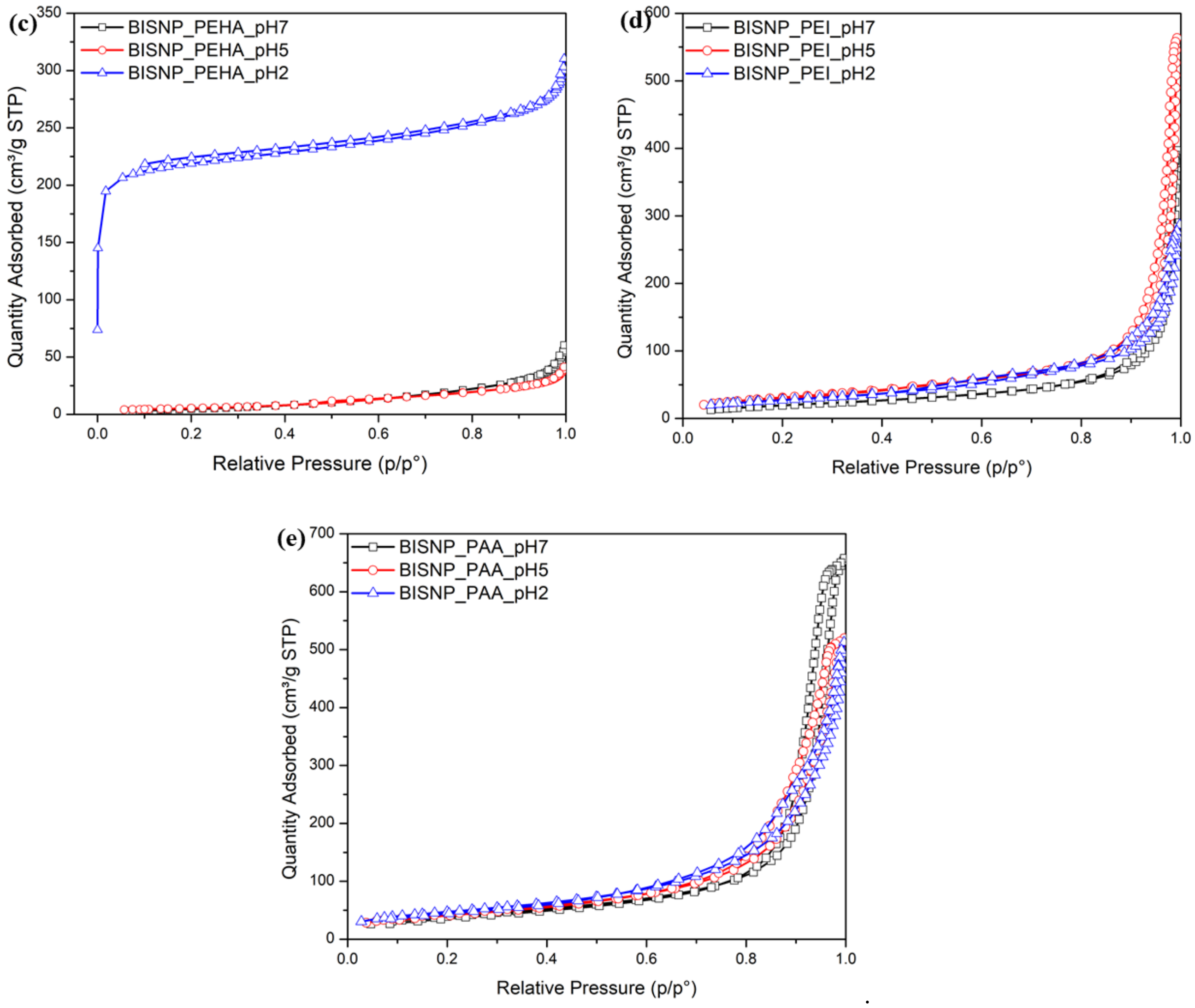

Figure S2. Different BIS isotherms (**a**) BIS_DETA samples, (**b**) BIS_TETA samples, (**c**) BIS_PEHA samples, (**d**) BIS_PEI samples, (**e**) BIS_PAA samples.

In Figure S3 are shown the FTIR analysis. Figure S3a, the support prepared at pH 7 with DETA additive, show the characteristic signals of the amines (1440–1480 cm^{-1} CH bending, 1650–1600 cm^{-1} bending of primary amines, 3000–3330 cm^{-1} NH stretching of secondary amines) in addition to those of the silica (1100–1000 cm^{-1} Si–O–Si stretching, > 3500 cm^{-1} Si–OH stretching). The intensity of the additive bands decreased for supports synthesized at pH 5 and 2. In the supports produced by PAA (Figure S3b) at pH 2, the additive is not completely removed as confirmed by the bands at 1500–1450 cm^{-1} due the C–H bending.

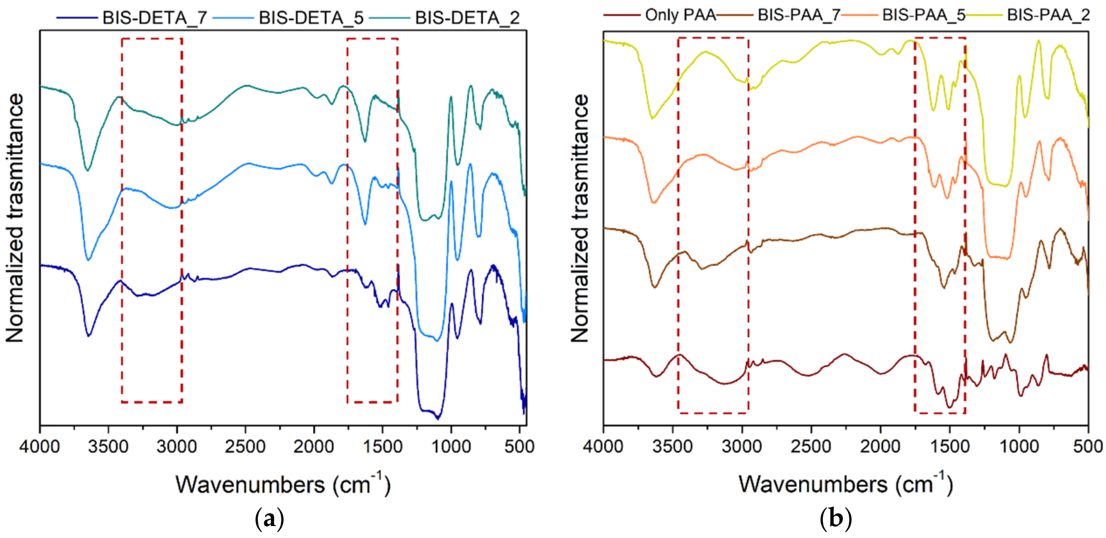

Figure S3. FTIR-DRIFT analysis of the (**a**) BIS-DETA, (**b**) BIS-PAA samples.

2. Protein and purification

In Figure S4 a are shown the surface potential of BSA (Figure S4a) and Cellulase (Figure S4b).

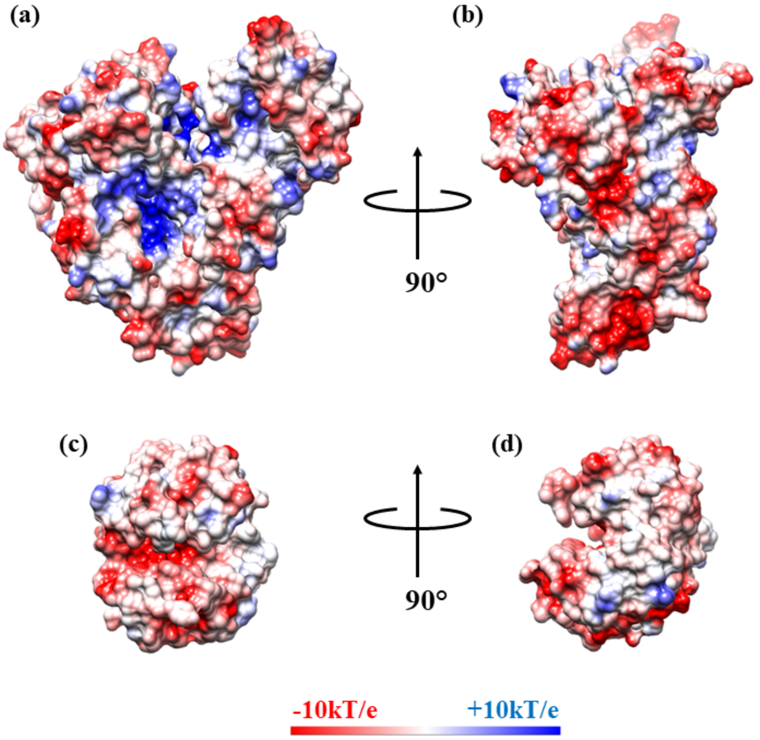

Figure S4. BSA and Cell_EG surface charge distribution. Molecular surface charge distribution of BSA, PDB id: 4F5S, (**a**,**b**) and cellulase, PDB id: 1KS5, (**c**,**d**). The potential distribution was generated and visualized with UCSF Chimera.

2.1. Cellulase purification

Cellulase from Aspergillus Niger (22178 Sigma Aldrich, Milan, Italy product) was dissolved in 50 mM Tris-HCl buffer at pH 7.4 to a final concentration of 10-20 mg/mL. and subsequently purified and analyzed with Size exclusion chromatography (SEC)

performed with ÄKTA pure 25 M system (GE Healthcare, Milan, Italy), using an HiLoad 16/600 Superdex 200 prep grade column in buffer Tris/HCl pH 7.4 at 50 mM (Milan, Italy).

The molecular weight of the protein was characterized by Sodium Dodecyl Sulphate Polyacrylamide Gel Electrophoresis analysis (SDS–PAGE), gel at 12% polyacrylamide. The protein samples were prepared by boiling for 10 min in the presence of denaturation buffer (Tris-HCl 0.5 M at pH 6.8, 10% SDS, 1% β-mercaptoethanol, Bromophenol blue solution at 0.12 % and 0.6 g of Glycerol). After denaturation, the protein samples were loaded on the polyacrylamide gel applying a voltage of 80 V and an intensity of electric current of 100 mA for 2 h. After migration, the gel was stained with Coomassie blue. Finally, the gel was analysed with GeneTools from Syngene software (version 08-3d.3 SynGene, Synoptics Ltd, Beacon House, Nuffield Road, Cambridge, CB4 1 TF, UK, 2000-2008) to calculate the molecular weight of the proteins. The Broad Range Standards (Bio-Rad, Milan, Italy) marker was used to characterize the molecular weight of the protein sample.

After size exclusion chromatography, a higher level of cellulase purity compared to the commercially available form is reached. Eluted protein showed the presence of two main peaks at ~68 mL of the elution volume and a smaller peak at around 75 mL, likely corresponding to the dimeric and monomeric form of the protein, respectively (Figure S5a). The protein showed an equilibrium between the two forms that are both catalytically active, as reported in the literature [1,2]. SDS-PAGE of cellulase protein prior and after the purification are shown in Figure S5b. After purification (lanes 3 and 4), the protein displayed a more intense band at 65 kDa, representing cellulase protein, and an overall decrease of the intensity of other contaminating bands.

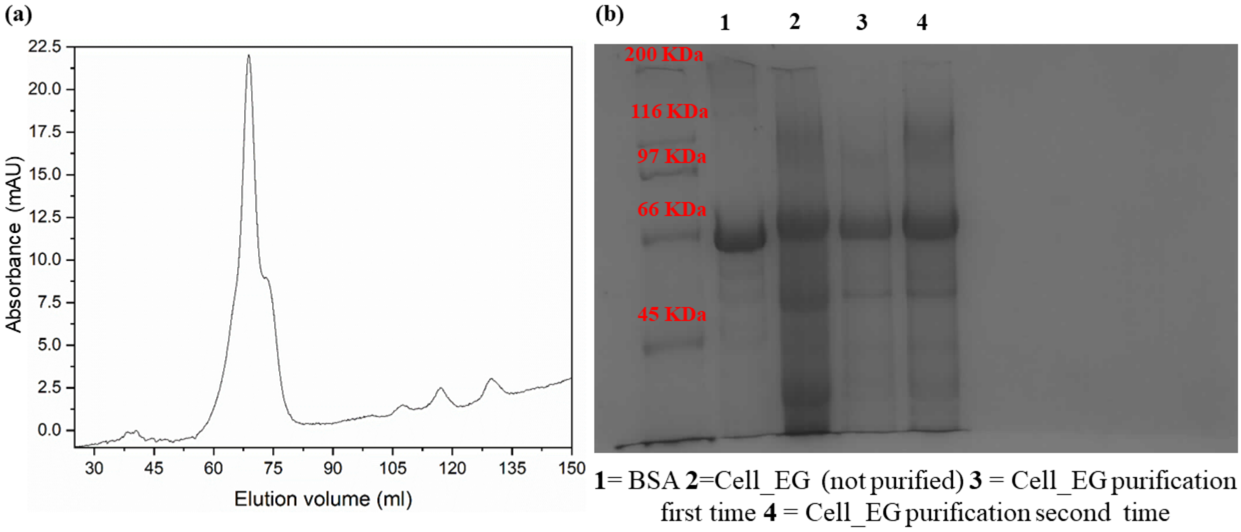

1= BSA **2**=Cell_EG (not purified) **3** = Cell_EG purification first time **4** = Cell_EG purification second time

Figure S5. (**a**) Chromatogram of the Cell_EG after second time purification, **(b)** SDS-PAGE gel of the Cell_EG after first and second time purification.

3. Analysis of the loading protein for confinement systems

The quantification of the proteins after different confinements was performed using the Brandford assay using different concentrations of BSA protein (from 1.2 mg/mL to 0.1 mg/mL) for the construction of the calibration curves (see Figure S6).

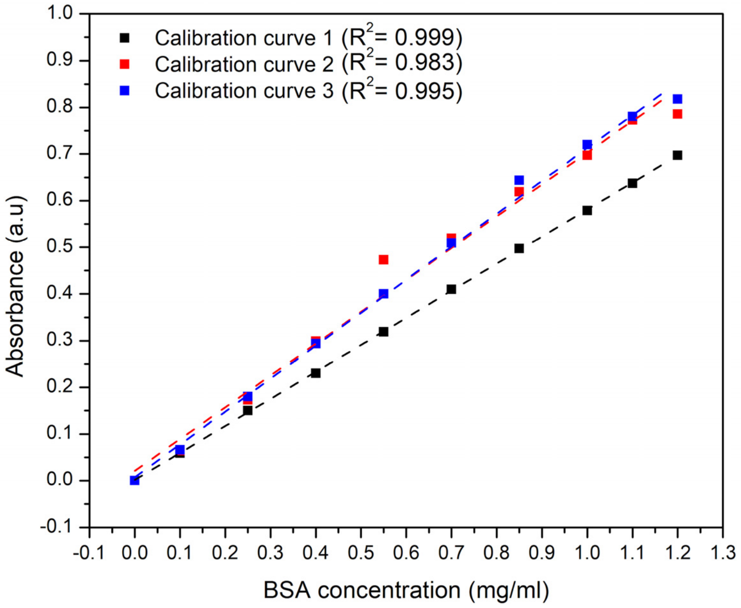

Figure S6. Calibration curves for the Brandford assay.

3.1. Adsorption system

In Tables S2 and S3 are summarized the samples, some morphological features and the loading efficiency for BSA and Cell_EG.

Table S2. Morphological features and BSA loading for different BIS systems.

Sample*	Surface area (m^2/g)	Pore volume (cm^3/g)	Loading efficiency (%) **
	**BSA Protein**		
BIS_DETA_7@BSA	32 ± 4.80	0.043 ± 0.01	2.79 ± 0.85
BIS_DETA_5@BSA	266 ± 40	0.15 ± 0.02	Negligible
BIS_DETA_2@BSA	402 ± 60.34	0.75 ± 0.10	14.55 ± 0.33
BIS_TETA_7@BSA	24 ± 3.63	0.044 ± 0.01	27.42 ± 10.50
BIS_TETA_5@BSA	211 ± 31.72	0.18 ± 0.02	1.34 ± 0.44
BIS_TETA_2@BSA	306 ± 46.00	0.19 ± 0.03	1.66 ± 1.47
BIS_PEHA_7@BSA	19 ± 2.80	0.044 ± 0.01	26.07 ± 1.63
BIS_PEHA_5@BSA	20 ± 3.00	0.18 ± 0.03	7.10 ± 2.04
BIS_PEHA_2@BSA	672 ± 100.85	0.19 ± 0.03	Negligible
BIS_PEI_7@BSA	75 ± 11.30	0.13 ± 0.02	71.66 ± 4.58
BIS_PEI_5@BSA	117 ± 17.65	0.19 ± 0.03	52.16 ± 22.21
BIS_PEI_2@BSA	100 ± 15.00	0.18 ± 0.02	Negligible
BIS_PAA_7@BSA	145 ± 21.75	0.4 ± 0.06	32.00 ± 3.12
BIS_PAA_5@BSA	159 ± 23.85	0.45 ± 0.07	52.69 ± 3.59
BIS_PAA_2@BSA	170 ± 25.50	0.41 ± 0.06	71.66 ± 4.58

* For each sample, the measurement was performed three times; ** The loading efficiency is measured after 3 washing.

Table S3. Morphological features and cellulase loading for different BIS systems.

	Cellulase protein		
**Sample ***	**Surface area (m^2/g)**	**Pore volume (cm^3/g)**	**Loading efficiency (%) ****
BIS_DETA_2@Cell_EG	402 ± 60.34	0.75 ± 0.10	Negligible
BIS_TETA_7@Cell_EG	24 ± 3.63	0.044 ± 0.01	10.4 ± 0.8
BIS_PEI_7@Cell_EG	75 ± 11.30	0.13 ± 0.02	4.9 ± 1.5
BIS_PAA_2@Cell_EG	170 ± 25.50	0.41 ± 0.06	28.3 ± 1.0

* For each sample, the measurement was performed three times; ** The loading efficiency is measured after 3 washing

3.2. Entrapment system

In Table S4 are reported the total loading efficiency for BSA and cellulase using the entrapment confinement is performed with bioinspired method.

Table S4. Different additives used and protein loading for entrapment method.

**Sample ***	**Additives**	**Loading efficiency (%) ****
BIS_DETA@BSA	DETA	50.6 ± 13.1
BIS_TETA@BSA	TETA	16.6 ± 2.1
BIS_PEHA@BSA	PEHA	10.5 ± 5.3
BIS_PEI@BSA	PEI	6.4 ± 2.4
BIS_PAA@BSA	PAA	87.2 ± 7.2
BIS_DETA@Cell_EG	DETA	5.35 ± 2.4
BIS_TETA@Cell_EG	TETA	16.0 ± 1.6
BIS_PAA@Cell_EG	PAA	34.3 ± 6.7

* For each sample, the measurement was performed three times; ** The loading efficiency is measured after 3 washing.

4. DNS assay for cellulase activity

4.1. DNS reagent preparation

The DNS reagent was prepared following the work of T. K. Ghose et.al.[3] In a falcon tube, 3,5-Dinitrosalicylic acid (0.05 M) was dissolved in a 50 mL of water and NaOH (0.55 M) solution and mixed until completely dissolved obtaining a yellow-orange colour solution. An amount of potassium sodium tartrate tetrahydrate (1.2 M), phenol (0.1 M), and sodium metabisulfite (0.05 M) were added to the solution and mixed at room temperature for 30 min.

4.2. Calibration and reducing sugar calculation

The cellulose activity was quantified by DNS method that consists of a redox reaction between the 3,5- dinitro salicylic acid (DNS) and the reducing sugars to produce the 3-amino,5-nitrosalicylic acid (orange-red) and gluconic acid as shown in the Figure S7. The intensity of the orange-brown color was analyzed by UV-visible at 500 nm.

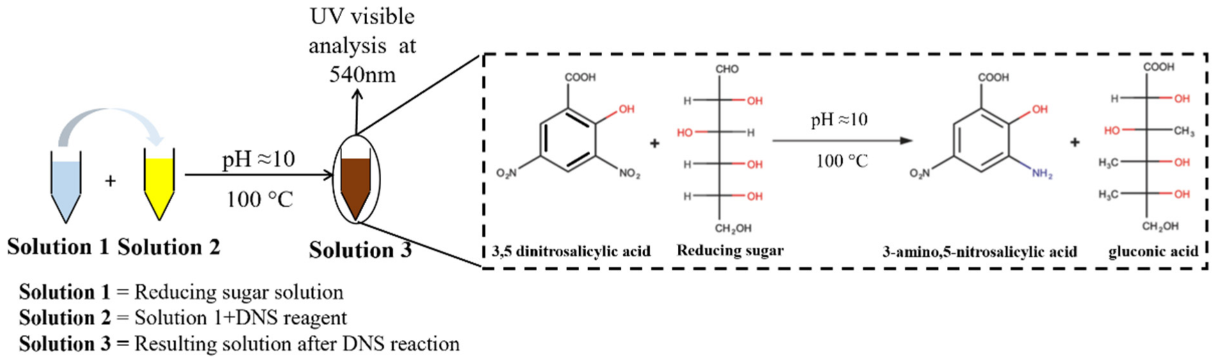

Figure S7. Scheme of the reducing sugar process by DNS method.

The quantification of the reducing sugars by DNS method was performed using a calibration curve constructed with different glucose concentration as shown in Figure S8.

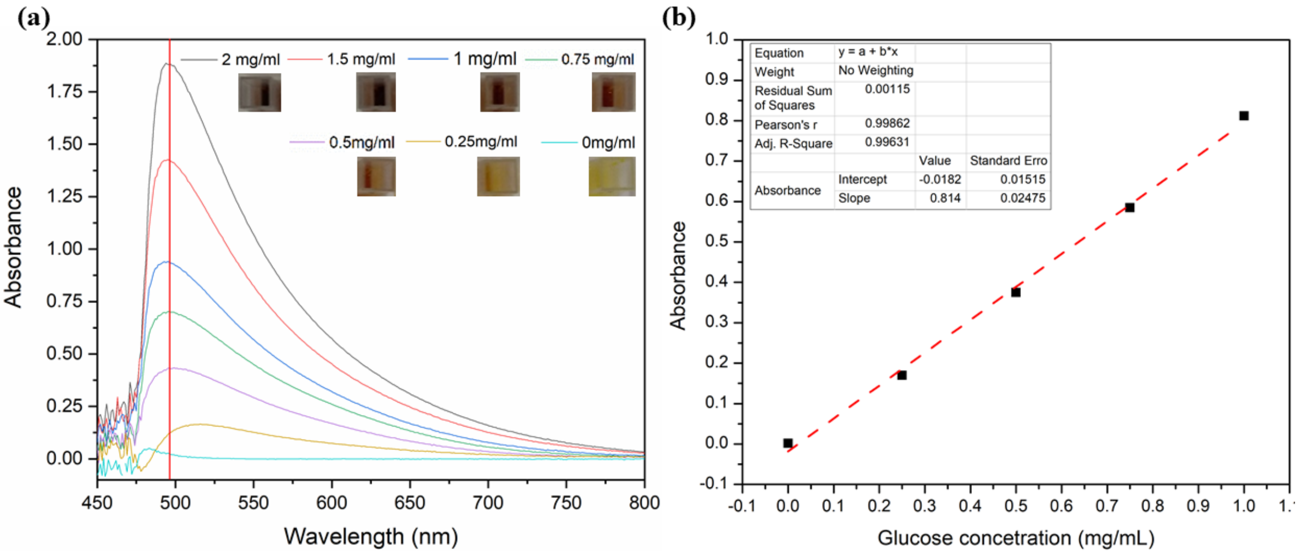

Figure S8. Calibration curve (**a**) Spectrum after DNS reaction with glucose standard (**b**) Construction of the calibration curve.

Table S5 summarizes the samples, the confinement strategy, the loading efficiency, and the relative activity. The activity test of the cellulase confinement was performed on the best samples of both adsorption and entrapment strategies.

Table S5. Loading efficiency (%) and relative activity (%) for both cellulose adsorbed and entrapped.

Samples	Confinement Strategy	Loading efficiency (%)*	Relative activity (%)*
BIS-PEI_7@ Cell_EG	Adsorption	4.9 ± 1.5	22.0 ± 0.5
BIS-TETA_7@ Cell_EG		10.4 ± 0.8	22.5 ± 0.1
BIS-PAA_2@ Cell_EG		28.3 ± 0.50	54.7 ± 0.7
BIS-DETA@ Cell_EG	Entrapment	5.35 ± 2.4	negligible
BIS-TETA@ Cell_EG		16.0 ± 1.6	43.3 ± 10.8
BIS-PAA@ Cell_EG		35.24 ± 5.2	90.4 ± 10

* For each sample, the measurement was performed three times.

References

1. Lübeck, M. *Cellulases:method and protocols*; Springer, Humana Press: New York, NY, USA, 2018.
2. Taipakova, S.M.; Smekenov, I.T.; Saparbaev, M.K.; Bissenbaev, A.K. Characterization of Aspergillus niger endo-1,4-β-glucanase ENG1 secreted from Saccharomyces cerevisiae using different expression vectors. *Genet. Mol. Res.* **2015**, *14*, 6439–6452.
3. Ghose, T.K. Measurement of Cellulase Activities. *Pure Appl. Chem.* **1987**, *59*, 257–268.
